# Reproductive Health Experiences Shared on TikTok by Young People: Content Analysis

**DOI:** 10.2196/42810

**Published:** 2023-11-13

**Authors:** Isha Nair, Sophia P Patel, Ashley Bolen, Samantha Roger, Kayla Bucci, Laura Schwab-Reese, Andrea L DeMaria

**Affiliations:** 1 School of Health Sciences Purdue University W Lafayette, IN United States; 2 Department of Biological Sciences Purdue University W Lafayette, IN United States; 3 School of Nursing Purdue University W Lafayette, IN United States; 4 Department of Public Health Purdue University W Lafayette, IN United States

**Keywords:** TikTok, social media, reproductive health, women's health, health outcome, content analysis, health information, sexual health, web-based information, COVID-19, health message

## Abstract

**Background:**

TikTok is a popular social media platform that allows users to create and share content through short videos. It has become a place for everyday users, especially Generation Z users, to share experiences about their reproductive health. Owing to its growing popularity and easy accessibility, TikTok can help raise awareness for reproductive health issues as well as help destigmatize these conversations.

**Objective:**

We aimed to identify and understand the visual, audio, and written components of content that TikTok users create about their reproductive health experiences.

**Methods:**

A sampling framework was implemented to narrow down the analytic data set. The top 6 videos from each targeted hashtag (eg, #BirthControl, #MyBodyMyChoice, and #LoveYourself) were extracted biweekly for 16 weeks (July-November 2020). During data collection, we noted video characteristics such as captioning, music, likes, and cited sources. Qualitative content analysis was performed on the extracted videos.

**Results:**

The top videos in each hashtag were consistent over time; for example, only 11 videos appeared in the top 6 category for #BirthControl throughout the data collection. Most videos fell into 2 primary categories: personal experiences and informational content. Among the personal experiences, people shared stories (eg, intrauterine device removal experiences), crafts (eg, painting their pill case), or humor (eg, celebrations of the arrival of their period). Dancing and demonstrations were commonly used in informational content.

**Conclusions:**

TikTok is used to share messages on myriad reproductive health topics. Understanding users’ exposure provides important insights into their beliefs and knowledge of sexual and reproductive health. The study findings can be used to generate valuable information for teenagers and young adults, their health care providers, and their communities. Producing health messages that are both meaningful and accessible will contribute to the cocreation of critical health information for professional and personal use.

## Introduction

### Background

Social media platforms allow users from various age groups to share and communicate information in an accessible manner. Prevalent social media platforms, such as Facebook, Instagram, Twitter, and TikTok, differ in layout and structure [1]. For instance, Twitter derives content from text; Instagram, from images; and Facebook, from a combination of both [1]. The increasing popularity of these platforms has created the potential for the spread of sensitive and stigmatizing topics regarding reproductive and sexual health promotion [2]. The public chooses the platform that best suits their needs based on how the layout and structure fit the context. Another factor is the stratification of social media platform users by age; younger people appear to prefer different social media platforms than those from previous generations. For example, a recent poll of 3000 Americans showed that 26% of Generation Z members chose TikTok as the app they would choose to use forever if they could only use one app, making it the top response for the entire age group [3]. This is in considerable contrast to older generations, which chose Facebook as their favorite by far (51% of all other ages of social media users) [3]. The relatively new platform TikTok launched in China in 2016 and went worldwide in 2018 with up to 800 million active users following the COVID-19 pandemic and global lockdowns [4]. TikTok, which allows users to create 15-second to 3-minute–long videos, has approximately 37 billion video views each month [5]. The platform is most popular among users aged between 13 and 24 years [5]. However, during the COVID-19 pandemic, some health care professionals and the World Health Organization turned to TikTok to facilitate the spread of reliable public health information [5].

### Digital Storytelling

Digital storytelling refers to how the public shares their story through various forms of media; using social media to do this has become popular. People often use social media to share their reproductive health experiences, whether they are stories of sexual assault, political statements, or health-related ordeals [2]. From #FreeThePill to #MyBodyMyChoice, early narratives being shared were crucial to large movements. One of the most well-known examples of such prominent social media movements is #MeToo. The phrase was initially used on the social media platform MySpace in 2006, but in 2017, it became very popular on Twitter, igniting the movement [6]. The goal was to promote, support, and reduce the stigma around sharing experiences of sexual assault. The hashtag quickly went viral, thrusting the topic into the spotlight [7]. Since then, the way reproductive health experiences are shared on the web has changed, with social media becoming a more prominent way to contribute to the narrative.

People find new, unique ways to share their stories every day, especially during the COVID-19 pandemic when they felt most isolated. This makes users feel more connected to each other and the world [8]. TikTok’s growing popularity has made it an ideal platform for young adults to share their experiences in a quick yet far-reaching manner [9], allowing users to share their health experiences uniquely through videos [10]. Even before TikTok, many social media users relied on platforms such as Twitter, YouTube, Facebook, and Instagram to share various reproductive health experiences [10-14]. For example, many nonprofessionals have shared overwhelmingly positive posts regarding intrauterine devices (IUDs), in contrast to the posts using more negative keywords from health care organizations and far more negative posts by law organizations, highlighting pain, issues with insertion and removal, and other side effects [11,15]. However, among the YouTube posts regarding IUDs, approximately one-third of the IUD testimonials contained inaccurate information that could skew a viewer’s opinion on IUDs and other contraceptives [13]. Miscarriage has also been discussed on social media, particularly on Instagram. The trending hashtag #IHadAMiscarriage provided women a way to cope and find support [12]. Abortion is another popular topic that has been discussed on social media. Through the popular hashtags #StandWithPP, #ShoutYourAbortion, and #NotoriousRBG, users can show their support and help normalize abortions [14]. These platforms have similar digital narratives; however, TikTok’s unique user interface and content-sharing methods are drawing attention. The platform exclusively shares videos and does so continuously, using an algorithm to ensure that each individual is seeing content curated specifically for them and adapting the stream accordingly [10,16]. On the basis of the content that viewers engage with the most, they receive similar videos [17]. Videos that are proven to be successful by some viewers will be pushed to others who might like similar content [16]. A study by Li et al [18] investigated the different video formats and types that TikTok provided specifically related to COVID-19 pandemic–related content and how other video formats and types contributed to user engagement. These video formats included video length, subtitle, text, spoken language, captions, and music, whereas the video types included whether they provided acting, an animated infographic, documentary, news, oral speech, a pictorial slideshow, or a TikTok dance [18].

Many popular platforms such as Facebook, Instagram, and Twitter focus predominantly on text or still images [10]. In contrast, the content on TikTok, often includes more detail with text, music, a continuous stream of content, and response videos to original TikTok content [10]. TikTok also allows users to combine several layers of overlapping content to create a unique narrative that differs from that of other platforms [19]. In addition, TikTok is trendiest among young people, especially in India, the United States, and Turkey, the countries with the largest monthly user base [20]. In fact, 41% of all TikTok users are aged 16 to 24 years, and in the United States, the top user ages fall between 18 and 24 years (42%) and 13 and 17 years (27%) [20]. Most TikTok users are young people, so the way content is spread on the platform conveys much about how young people interact with the information on it. Because TikTok rarely features content from familiar users, this narrative empowers the younger demographic to connect with new individuals in a more accessible way than other well-known social media platforms [19]. Although it is understood that TikTok’s digital narrative differs from other platforms, there is currently minimal research on how reproductive health content is spread on this growing platform.

### Social Media in Health Care

The growing prevalence of social media has also provided health care professionals with a platform to effectively share health information and public health interventions with over 1 billion users worldwide [21]. Because it is such a prominent way that important information is being spread, it is essential to understand how younger people, especially those disproportionately impacted by negative health outcomes (13-24 years), use the app to talk about reproductive health and what topics most interest them. In the same way in which people create videos and partake in trends to share health information involving periods, birth control, infertility, and more, users also make TikTok content about all types of relationships: romantic, platonic, familial, and professional [9,22]. As relationships play a large role in how people view topics and make decisions about topics such as reproductive and sexual health, it is an important aspect to study. However, there is not much research about how relationships are discussed on TikTok. This study can provide crucial pilot data for future research and program development proposals that use technology to reach young people. Analyzing teenagers’ and young adults’ behavior on TikTok when discussing and engaging in reproductive health experiences can not only generate valuable information for them, their health care providers, and their communities but also help produce health messages that are both meaningful and accessible. The insight we gained from this investigation can allow for contributions to the cocreation of critical health information for professional and personal use. Therefore, this study aims to identify and understand the content TikTok users are sharing about their everyday reproductive health experiences and the audio, visual, and written techniques they use to do so.

## Methods

### Data Collection

This study used an approach based on the principles of program science to inform the planning, implementation, and evaluation of complex health interventions [23]. This iterative approach consists of 3 primary phases, providing a framework for moving research and practice forward when an evidence-driven solution does not exist. The strategic planning stage focuses on developing a solid understanding of the current situation to enable informed decisions, the program implementation stage focuses on developing and refining the specifics of the program, and the program management and evaluation stage uses an ongoing and iterative quality improvement approach based on the evolution of the program. Future strategic planning that further refines the program will be informed by the knowledge generated through the program management and evaluation phase. This study focused primarily on the strategic planning phase, in which an understanding of the current situation was developed to aid future program development and implementation.

A qualitative content analysis approach was used, consistent with our prior research [24-29]. TikTok has ≥1 billion active users. Therefore, an appropriate sampling framework was identified to reduce the scope of the analytic data set. At the beginning of the project period, a number generator was used to identify 2 random days for each week of data collection. On these identified days, the top 6 videos, in terms of viewer engagement, for several different hashtags were extracted. TikTok’s algorithm implies that the top videos in these hashtags are the videos young people interact with the most. This video extraction occurred biweekly for 16 weeks from July to November 2020 in the United States. To analyze how creators present information on reproductive health, some popular hashtags were identified and selected based on the study’s research questions, a preliminary review of hashtag use, and alignment with the funding mechanism: #MyBodyMyChoice (2.2 billion views), #BirthControl (3.5 billion views), #PeriodProblems (1.4 billion views), #OBGYN (4.0 billion views), #WomensHealth (5.9 billion views), and #FreeThePill (1.4 million views). The hashtags #LoveYourself (21.1 billion views), #CoupleGoals (236.2 billion views), #BestFriend (81.1 billion views), and #Relationship (148.4 billion views) were studied to analyze how relationships were represented on TikTok. Although these hashtags may have multiple potential permutations, the versions that were most viewed were those used.

### Data Analysis

After data collection was completed, the analysis phase was conducted. As with prior research, a codebook to analyze the TikTok videos was created using a content analysis framework [30-33]. Content analysis is a term used to describe a range of qualitative or quantitative methodologies. For this project, conventional content analysis, as defined by Hsieh and Shannon [31], was used where the coding frame was directly derived from the text (Multimedia Appendix 1). This approach is used frequently to compensate for the limited research on a subject [31]. The code-generation process was based on the adaptation of Schreier [33] on grounded theory. We began the analysis by reviewing the extracted TikTok videos. During a second review of the TikTok posts, notes were taken on emerging concepts with a particular focus on concepts that repeated or diverged between posts, making a note of recurring songs (if music was used), and the number of likes, views, comments, and shares on each post, all metrics of viewer engagement. In addition to the content, the length of the posts, whether said content was typed or spoken and whether it was part of a duet or challenge, was also recorded. This framework ensured broad insight into what users were creating, engaging with, and reproducing. A draft codebook was developed based on these notes and discussed among the entire research team to apply shared perspectives and finalize the codebook based on consensus. Once the codebook was finalized, it was applied to 5% of the available TikTok posts. On the basis of this pilot study, the codebook was revised and implemented to define the codes and categories. This process continued until the team determined that the codebook captured and defined the various domains and theoretical propositions under study and the team agreed on the applied codes [29,34]. As coding progressed, the team communicated regularly to discuss coding and resolved any uncertainties via discussion.

Then, segments were reviewed in relative isolation after the codes were applied. The segmentation process described by Schreier [33] was followed. In general, 1 study found that the use of video made HIV/AIDS content more engaging and more emotionally impactful [35]. As TikTok content come in video form, visual content, whether written or physical, is essential for a video’s success. However, according to the platform itself, audio is just as important if not more. TikTok notes that 88% of its viewers consider audio vital to the experience and 50% say that music makes content more engaging and energizing [36]. It was also found that recall increases by 8 times the baseline when distinctive sounds are used [36]. The audio, visual, and written aspects were analyzed separately for each video. The audio segment was coded by IN, the visual segment was coded by AB, and the written segment was coded by SPP. For some codes, patterns and themes were identified in the content to describe the results qualitatively. For other codes, categorical variables (eg, primary topic and the sentiment of comments) were created, allowing the basic characteristics of the analytic data set to be summarized. The codes used for the audio aspect of each video involved topic, goal, tone, speed, duration, perceived gender of the speaker, points of view, the number of audio sources used, and the type of audio source used. It must also be noted that tone is a subjective code. Although insightful, it depends on the coders’ perceptions and may be considered less reliable for that reason. Similarly, the speed of speech was also judged comparatively by the coders. For the visual aspect of each video, things such as the attire of the user, content type, methods and features used, number of people, perceived gender, presence of the user, props used, TikTok user, and video location. Similarly, for the written aspect of each video, the number of individual text boxes, text duration, the color of text, font of the text, goal of the text, caption content, number of hashtags present in the caption, hashtag content, presence of “for you page” hashtags, and the overall importance of writing in the video to understand the TikTok was analyzed. This process allowed us to identify differences in patterns and themes based on the characteristics of the conversations in the TikTok videos.

### Ethics Approval

This study was approved by Purdue University’s institutional review board (IRB-2020-803).

## Results

A few key aspects comprise every piece of TikTok content—audio, visual, and written components. The results demonstrate the most popular ways users incorporate these essential aspects, as well as how health care professionals and other health practitioners may best use them to discuss this content with their patients and community members. In terms of content (Table 1), there was extensive overlap between different themes among the reproductive health hashtags observed. This caused 10 of the 100 videos to be duplicates. Most of these (7/100, 70%) came from the hashtag #WomensHealth. This resulted in 90 videos being coded. Numerous types of reproductive health topics were shared on this platform. Almost one-quarter of the videos (22/90, 25%) discussed relationships, making this the most discussed topic. The next most common topics were equal in prevalence—birth control and hygiene in health, with 15% (13/90) of videos being about them. Many videos were about periods or political situations (eg, abortion and punishments for sexual assault). The remaining topics occurred less frequently.

**Table 1:** Content results (n=90).

Topic	Occurrence, n (%)
Relationships	22 (25)
Birth control	13 (15)
Hygiene and health	13 (15)
Periods	11 (12)
Political situations	12 (13)
Birth and pregnancy	4 (4)
Sex education	2 (2)
Assault and safety	1 (1)
Body positivity	1 (1)
Other	11 (12)

### Audio Aspect of Videos

There are several ways in which audio (Table 2) may convey a specific message on TikTok. Users make many choices in terms of audio to better suit the purposes of their TikTok. Of the 90 videos coded, there were some common trends. For example, popular videos seemed to have only one speaker at a time when disseminating reproductive health information. Most videos (66/90, 73%) came from a single individual speaking or music by a female singer. While the remaining videos may have multiple audio sources, approximately 99% (89/90) of the videos included only one perspective—no duets or stitches (ie, methods of adding to and interacting with a video that has already been made).

**Table 2:** Audio results (n=90).

Codes and subcodes		Occurrence, n (%)
**Type of audio**		
	Just music	36 (40)
	Just speaking	43 (48)
	Both music and speaking	11 (12)
**Perceived speaker or singer**		
	Singular female individual	62 (68)
	Both female and male individuals	17 (19)
	Multiple female individuals	6 (7)
	Singular male individual	4 (4)
	Multiple male individuals	1 (1)
**Perceived tone**		
	Comedic	32 (36)
	Enthusiastic	25 (28)
	Angry	8 (9)
	Sad	5 (6)
	Informative or monotonous	20 (22)
**Speed**		
	Fast	12 (13)
	Slow	42 (47)
**Duration**		
	Whole TikTok	87 (97)
	≥75% of TikTok	2 (2)
	25%-50% of TikTok	1 (1)
**Purpose of audio**		
	Tell a story	19 (21)
	State an opinion	8 (9)
	Informational	13 (14)
	Provide additional context or commentary	14 (16)
	Participate in a trend	11 (12)
	No connection or background	20 (22)
	Other	5 (6)
**Number of audio sources**		
	1	88 (98)
	2	2 (2)
**Multiple points of view**		
	Duet or stitch	1 (1)
	None	89 (99)

There is slightly more variability with the rest of the results. For example, 48% (43/90) of the collected videos only had speaking as the audio source while 40% (36/90) only had music. The remaining 12% (11/90) had both. Of the 54 videos that contained speaking, there were variations in tone and speed. Of the speakers, 47% (42/90) spoke noticeably slower than the remaining speakers. The sample of videos analyzed contained a broad range of topics and used different tones. For example, 36% (32/90) of the coders perceived the videos as comedic and 28% (25/90), as enthusiastic. Some participants (20/90, 22%) seemed to have no emotion and a more informative, monotonous tone. The remaining videos contained intense emotions such as sadness or anger. TikTok creators used audio for many other purposes. From the data collected, some videos had audio entirely unconnected to their message (20/90, 22%), while others were telling a story (19/90, 21%) or stating an opinion (8/90, 9%). Other prominent purposes were to provide information, following a TikTok trend or using their audio as a supplement that provided additional context and commentary on what they were trying to say. Of the videos coded, 6% (5/90) were more unique to the reproductive health realm, such as debating specific political issues, and had audios that could not be characterized as having any of the purposes listed earlier.

### Visual Aspect of Videos

The visual content (Table 3) in each of the 90 TikTok videos was also analyzed and collected to evaluate how influencers presented reproductive health information to TikTok viewers. After compiling the data, the types of videos that were popularized in terms of visual content could be seen. A prominent feature in all videos was the presence of text at some point. Text was shown in the video 43% (39/90) of the time. Text was coded if there were visual words on the video. Captions were not considered as text. Other codes with common trends that displayed popular creator choices were as follows: the user’s attire, number of people, perceived gender, TikTok user type, video location, and features used. Looking at the user’s attire, most (75/90, 83%) individuals did not dress in medical uniforms (eg, scrubs or a laboratory coat). Regarding the type of TikTok users, 80% (72/90) were general users: ordinary people who were neither famous nor presenting as medical professionals. Nearly one-fifth (17/90, 19%) of the participants were medical professionals. In the videos, it was common to see only 1 individual present (62/90, 69%). Regarding video location, more than half (51/90, 57%) were filmed in what appeared to be a personal residence. No videos were located in a hospital, and the remaining videos were in other locations, such as an office or a car.

**Table 3:** Visual results (n=90).

Codes and subcodes		Occurrence, n (%)
**Presence of text**		
	Present	39 (43)
	Not present	51 (57)
**Attire**		
	Casual	48 (53)
	Scrubs	13 (15)
	Laboratory coat	1 (1)
	Combination	1 (1)
	Other	27 (30)
**Number of individuals present**		
	1	62 (69)
	Other	28 (31)
**Type of creator**		
	General	72 (80)
	Medical professional	17 (19)
	Celebrity	1 (1)
**Location**		
	Personal residence	51 (57)
	Hospital	0 (0)
	Office	4 (4)
	Outdoors	14 (16)
	Other	21 (23)

### Written Aspect of Videos

Another video aspect analyzed was the written content (Table 4) presented. Of the videos analyzed, 41% (37/90) had no textboxes and the rest had varying numbers of textboxes. The color and font of the text present in the TikTok content also varied, with no clear trends. Overall, 27% (24/90) of the videos used text as a description for audio or visuals. The rest used text for different reasons, such as to reply to comments, to label visuals, and subtitles, to name a few. Most videos used at least 1 textbox, caption, or hashtag as a secondary form of communication after visual and audio, and over half of the videos used textboxes, where their primary use was to describe the audio or visuals in the video. Captions were another way to present information. Captions were used to further describe the video content in 48% (44/90) of videos. An additional 26% (23/90) of captions presented information on health-related topics such as abortion, birth control, friendship, menstruation, relationship with self, romantic relationship, sex, and other health content. Moreover, 8% (7/90) of the captions presented hashtags only, while the rest had completely unrelated captions that served the purpose of giving credit to trend creators or asking their viewers to interact. Hashtags were present in nearly all (83/90, 92%) of the videos, with most (66/90, 73%) containing ≥5 hashtags. In 28% (23/90) of the videos, video creators used hashtags that were directly related to their video content. In 57% (51/90) of the videos, the videos contained hashtags that were both related and unrelated to the content. In addition, less than half of the hashtags in the captions were “#ForYouPage,” “#ForYou,” or “#FYP.”

**Table 4:** Written results (n=90).

Codes and Subcodes		Occurrence, n (%)
**Textboxes present**		
	No textboxes	37 (41)
	1-4 textboxes	25 (28)
	≥5 textboxes	28 (31)
**Color and font**		
	Black or white font	31 (34)
	Colored font	22 (24)
	Both black or white font and colored font	7 (8)
	Default font	48 (53)
	Multiple fonts	3 (3)
	Typewriter font	1 (1)
**Purpose of text**		
	Reply to comments	2 (2)
	Subtitles	6 (7)
	Description for audio or visuals	24 (27)
	Clarify myths	2 (2)
	Give personal advice	2 (2)
	Label visuals	4 (4)
	Provide information	10 (11)
**Purpose of captions**		
	Describe content further	44 (48)
	Ask viewers to interact	4 (4)
	Give credit to creator of trend	1 (1)
	Present information unrelation to health content	11 (12)
	Hashtags only	7 (8)
	Present information related to health topics	23 (26)
**Hashtags present**		
	Present	83 (92)
	Not present	7 (8)
**Number of hashtags**		
	≥5	66 (73)
	≤5	24 (27)
**Relation of hashtags to reproductive health content**		
	Hashtags related to one topic addressed in video	23 (28)
	Hashtags related to multiple topics addressed in video	23 (26)
	Hashtags both related and unrelated to content	51 (57)

## Discussion

### Principal Findings

This study aimed to better understand the distinct components of content that TikTok users incorporate to create reproductive health-related information and share their experiences on this social media platform. The results demonstrate that there were some key aspects of videos that appeared to contribute to success. Although the number or style of the textboxes seems insignificant, audio appears to be essential for success. In addition, having some kind of tone or emotion likely increases engagement, and general content creators seem to connect better and have more reach with the public than a health care provider does.

Several reproductive health topics were discussed by users. Although some videos did not fall under any of these categories, the most popular subjects, in order of decreasing popularity, were relationships, birth control, hygiene and health, political situations (eg, abortion and punishments for sexual assault), periods, birth and pregnancy, education on sexual intercourse, assault and safety, body positivity. This suggests that these are the topics viewers are most curious about and the areas health care providers and other health practitioners might want to address when educating their patients and communities.

We assessed the role of audio, visual, and written approaches in the sharing of reproductive health-related information. In doing so, we found many commonalities among the most viewed videos. The common trends observed within the audio choices suggest a potential correlation between the way audio is used in TikTok and the video reach. Platforms such as Twitter, Instagram, and Facebook allow videos with audio but also often rely on pictures with no audio [10]. The fact that all videos on TikTok had some form of audio implies that audio may be essential to any TikTok’s success—something any user, including health experts, should remember when trying to use the platform to reach young people. In addition, most TikTok content had only 1 speaker, if any. They also mostly came from one perspective, with no stitches or duets. Although other videos across the platform commonly used the feature that allows you to add another video as a form of reply to continue a conversation, not one of the reproductive health videos we studied did so. This may be due to the sensitivity of some of the subjects, as well as the lack of universal knowledge on them. Popular videos used only one audio source at a time, such as a song or spoken content rather than both together, suggesting that more than one source may be too much of a distraction when trying to process the content in a meaningful manner.

Most videos appeared to have some kind of tone, such as anger, sadness, or excitement. They also had slower-paced spoken words. This is probably a tactic to connect with viewers more easily, and it seems to be something to which they respond well. Slower speech may be easier to understand and process, getting the message across more efficiently, but slow speech in conjunction with a monotonous tone would likely bore audiences who care more about what creators have to say when it emotionally impacts them. In fact, according to a study conducted by Holub [37] on the effects of intonation, it was found that monotony has a negative impact on audience comprehension. However, some TikTok content is purely informational. Although it might not make sense for those videos to use tone in an emotional manner, an enthusiastic tone or the inclusion of upbeat music and dancing goes a long way to making the video noticeable and therefore allowing it to deliver information to viewers.

Analyzing the visual content showed that content creators who wear casual clothing, film in a personal residence, and come off as general content creators rather than health care providers reach more TikTok users about reproductive health. It is possible that viewers find this information more credible when it comes from someone they can relate to and find these informal settings more comfortable. These factors are all things that providers can consider when creating an appealing environment to spread information in their videos.

Following Comp et al [10], we found that the written aspects of TikTok videos were used to either supplement audio and visual information or to guide viewers to audio and visual information rather than to serve as the primary form of communicating information to viewers during the COVID-19 pandemic. We found that the number or style of textboxes used in each video had no apparent pattern. The primary purpose of the captions was to guide the viewer and provide additional information. Overall, although written content seemed to be a popular tool to enhance the video and provide elaboration, video creators did not have a specific way to present written content.

Hashtags served an overall greater purpose in comparison with textboxes and captions. They connected viewers to a video so that they could learn about women’s reproductive and sexual health through the video’s primary forms of communication: audio and visual content. Furthermore, many of the findings of Zappavigna [38] and the conclusions of Herrman [39] on the use of hashtags were echoed in this study, where hashtags served a classificatory function, providing a way for viewers to identify content based on a specific topic, as opposed to presenting additional information on the content as captions did. Hashtags also allowed creators to connect themselves and their content to other TikTok creators and viewers who may share similar stories, content, or opinions on reproductive and sexual health. The use of multiple hashtags for one video led to some overlap in data collection. The occurrence of numerous hashtags could have been related to the creator’s desire to increase the searchability of their content and increase their range of audience, which should be considered by experts and educators when creating videos and looking to increase reach.

### Limitations

Although this study provides insight into how information on reproductive health is shared on TikTok, it has its limitations. As discussed earlier, one of the things that makes studying TikTok so important is that it is constantly changing; therefore, some new aspects cannot be encompassed in this research. For example, when data collection began, the longest a TikTok video could be was 1 minute. Since then, an update to the app has increased the amount of time a video can run from 1 minute to 3 minutes. Thus, our research cannot examine how this change affects what are now the most popular videos under these hashtags. This could have a huge impact on popular creative choices and is an aspect we could not explore. There may be other similar changes that were not anticipated that continue to affect popular trends. Some of these codes are also quite subjective, such as the speed, tone, and perceived gender of the speaker, which depend on the coder’s perspective. Although these are vital aspects of each video that should be examined, the reliability of our study was affected by the lack of intercoding in each segment, a change that will be made for future studies.

This study and future studies could benefit from a larger sample size with data collected over a longer period. The level of overlap in our data decreased the number of videos analyzed. Although this says a lot about the popularity of certain visual or audio aspects of these videos, there was not necessarily enough material to explore things that varied more, such as written content, to the extent it could have been. Further analysis of a larger video sampling can provide more insight into the written content trends and provide more validity to the study.

Despite these limitations, this project is vital because it sheds light on how reproductive health information is shared among young people on a major social media platform. It focuses on exactly how reproductive health information is shared on TikTok, showing the great variety of topics shared but also how there are so many similarities in how that information is shared. Many trends were identified that can guide health care providers and other health practitioners to build a toolkit to better disseminate their information to a larger audience.

### Implications

TikTok is an expanding digital app that continuously engages more young adult users every day. It is a platform in which many content creators have access to different formatting tools to captivate viewers by spreading information regarding reproductive and sexual health topics. In the age of social media, TikTok is a tool to spread information that many people have already been engaging with. Even before TikTok emerged, there have been studies examining the impact of social media and its effect on the medical field.

One specific study by Omurtag and Turek [40] discussed how to provide guidance for reproductive health professionals and how to use social media to effectively disseminate information, specifically on infertility and in vitro fertilization. A key aspect they found that makes social media appealing to individuals is anonymity—individuals who engage on these platforms do not have to include any contact information, allowing for privacy [40]. Although both studies promote the use of social media by medical professionals, our study goes into depth specifically on what topics users interact with and how they view them, providing insight that medical professionals can use to make videos that are both meaningful and accessible to young adults. Our study was able to summarize the common aspects of videos that were appealing, such as having a casual setting and a creator perceived to be a woman as the main messenger. Relatability was key, and videos with these features overall showed a higher chance of being viewed.

The videos analyzed had specific hashtags related to reproductive health, including #MyBodyMyChoice, #BirthControl, #PeriodProblems, #OBGYN, #WomensHealth, and #FreethePill. Many of these topics deal with individuals’ personal stories or experiences. It is important to see what the users are drawn to understand how to best reach them regarding important information about women’s reproductive health. Future research could examine each specific hashtag. Individuals may respond uniquely to the content within each hashtag and its associated topics or create content using a specific methodology. The study by Li et al [18] found that for COVID-19 pandemic content, dancing videos were favored significantly more than any other method when presenting the information. This was not the case in our study (7/90, 7%), likely because our content did not include all COVID-19 pandemic content but rather reproductive health content shared at the height of the pandemic. Our study did, however, have similar findings on what other features users seem to engage with most. These interesting features included captions, hashtags, humorous tones, primarily visible human subjects, and some background audio. The features common to both studies allow a greater understanding of the information provided in the video and contribute to the increased engagement of users. The data collected in our study provide new information on what individuals engage with the most to guide health practitioners in creating impactful and viewable content. We found that some kind of tone, humorous or not, was helpful, and popular women’s health–themed hashtags were a major way to ensure a larger audience. Looking further at a specific topic in women’s health might allow deeper insight into what viewers engage best within each topic and allow professionals to provide meaningful information. Another area of research related to this is examining the methods of each video after observing the number of shares, likes, and comments.

### Conclusions

This study sought to understand how young people shared reproductive health information on TikTok, which was captured during the COVID-19 pandemic. Most videos presented content that was either helpful or relatable to the viewer. In addition, videos in which a single individual was dressed informally and videos that used audio for the entire duration were viewed more often. The written aspect of the videos was supplemental to the visual and audio content. If health care professionals present content in this way, young people will continue to be open and willing to learn about women’s reproductive and sexual health on social media platforms such as TikTok.

TikTok is a continuously growing platform. Currently, we are in the age of social media and many young people share and receive information, including information related to reproductive health. Health care professionals and other health practitioners have an opportunity to provide reproductive health information through social media, especially through TikTok. Because of this study, a guideline on how to make the most meaningful and engaging videos can be created. These guidelines may include the use of captions, hashtags, a humorous tone, a primarily visible female creator, and background audio. By following these guidelines, creators can develop and share videos that are meaningful and engaging to TikTok users worldwide.

## Appendix

Multimedia Appendix 1 Codes and subcodes determined for audio, visual, and written codebooks used to analyze video creation choices of TikTok users discussing reproductive and sexual health.

## Abbreviations

IUD: intrauterine device
