# Supplementary material for: Reproductive Health Experiences Shared on TikTok by Young People: Content Analysis
Source: JMIR Infodemiology. 2023 Nov 13;3:e42810. doi: 10.2196/42810 (PMC10682920; doi:10.2196/42810)
Supplement: Multimedia Appendix 1 [file infodemiology_v3i1e42810_app1.docx]

**APPENDIX A: Codebooks**

**Audio Codebook**

| **Codes** | **Sub-Codes** |
| --- | --- |
| Type of Audio |  |
|  | Just Music |
|  | Just Speaking |
|  | Both Music and Speaking |
|  | No Audio |
| Number of Audio Sources |  |
|  | 0 |
|  | 1 |
|  | 2 |
|  | 3 or more |
| Goal of Audio |  |
|  | Tells Story |
|  | States Opinion |
|  | Informational |
|  | Provides Additional Context or Commentary |
|  | Participates in Trend |
|  | No Connection/Background |
|  | Other |
| Duration |  |
|  | Throughout Whole TikTok |
|  | 25% of TikTok or Less |
|  | 25% - 50% of TikTok |
|  | 50% - 75% of TikTok |
|  | 75% of TikTok or More |
|  | No Audio |
| Tone |  |
|  | Enthusiastic |
|  | Comedic |
|  | Angry |
|  | Sad |
|  | Informational/Monotonous |
| Speed of Speech |  |
|  | Fast |
|  | Slow |
|  | No Speech |
| Multiple Points of View |  |
|  | Duets/Stitches |
|  | None |
| Perceived Number and Gender of Speaker(s) (Speech or Music) |  |
|  | Multiple Males |
|  | Multiple Females |
|  | Both Female and Male |
|  | Singular Female |
|  | Singular Male |
| Content |  |
|  | Birth Control |
|  | Birth/Pregnancy |
|  | Body Positivity |
|  | Hygiene/Health |
|  | Assault/Safety |
|  | Periods |
|  | Political Statement |
|  | Relationships |
|  | Sexual Intercourse |
|  | Other |

**Visual Codebook**

| **Codes** | **Sub-Codes** |
| --- | --- |
| TikTok User |  |
|  | General |
|  | Medical Professional |
|  | Celebrity |
| Content Type |  |
|  | Dancing |
|  | Demonstration |
|  | Speaking to Camera |
|  | Lip-Syncing to Music |
|  | Other |
| Props Used |  |
|  | Contraception (IUD, Pill Pack, etc.) |
|  | Menstrual Products (Period Cup, Tampon, etc.) |
|  | Anatomical Model |
|  | Other |
| Perceived Gender of Creator |  |
|  | Male |
|  | Female |
|  | Unseen on Camera |
| Presence of User (If Applicable) |  |
|  | Makeup |
|  | No Makeup |
| Attire |  |
|  | Casual |
|  | Dress/Fancy |
|  | Lab Coat/Scrubs |
| Number of People in Video |  |
|  | 0 |
|  | 1 |
|  | 2 |
|  | 3+ |
| Methods/Features |  |
|  | Stickers |
|  | Text |
|  | Green Screen |

**Written Codebook**

| **Codes** | **Sub-Codes** |
| --- | --- |
| Text Duration |  |
|  | Text Throughout Whole TikTok |
|  | Text for Less than Half of the Duration |
|  | Text for More than Half of the Duration |
|  | No Text |
| Number of Individual Text Boxes |  |
|  | 0 |
|  | 1 |
|  | 2 |
|  | 3 |
|  | 4 |
|  | 5 |
|  | 6 or More |
| Color of Text |  |
|  | Black and/or White |
|  | Colored Text |
|  | Both Black and/or White Text and Colored Text |
| Font |  |
|  | Default |
|  | Serif |
|  | Handwriting |
|  | Typewriter |
|  | Neon |
|  | Multiple Fonts |
| Goal of Writing |  |
|  | Act as Closed Captions |
|  | Provide Commentary |
|  | Dispel Myths |
|  | Tell a Story |
|  | Give Advice |
|  | Description of TikTok |
|  | Label Visuals |
| Importance of Writing to Understanding Content on Scale of 1-5 |  |
|  | 1.00 – Text is Inessential to Understanding Meaning of TikTok; Writing is Least Important Aspect of Communication |
|  | 3.00 – Writing is Equally as Important as Other Aspects |
|  | 5.00 – Text is Essential to Understanding Meaning of TikTok; Writing is Prominent Way of Communication |
| Number of Hashtags |  |
|  | 1 |
|  | 2 |
|  | 3 |
|  | 4 |
|  | 5 or More |
| Hashtag Content |  |
|  | Related to Content of TikTok |
|  | Only Unrelated Hashtags |
|  | Related and Unrelated Hashtags |
| Caption Content |  |
|  | No Caption (NO TRANSCRIPT PRESENT WITH VIDEO) |
|  | Ask Viewers to Interact |
|  | Caption Further Describes Content |
|  | Caption Gives Credit to Creator of Trend |
|  | Only Hashtags |
|  | Caption Related to Health Content of Video (Birth Control, Sex, Menstruation, Abortion, etc.) |
|  | Caption Unrelated to Health Content of Video |
| For You Page Hashtags |  |
|  | #fyp, #foryou, or #foryoupage |
|  | No For You Page Specific Hashtags |
